# PReS-FINAL-2107: In vitro investigation into mesenchymal stromal cells as a potential therapeutic in juvenile arthritis

**DOI:** 10.1186/1546-0096-11-S2-P119

**Published:** 2013-12-05

**Authors:** J Wienke, S De Roock, J Swart, A Martens, N Wulffraat, B Prakken

**Affiliations:** 1 UMC Utrecht, Utrecht, Netherlands

## Introduction

Mesenchymal stromal cells (MSC) are multipotent cells with an immune suppressive capacity. In the last decade the therapeutic application of these cells has been tested in inflammatory diseases like graft versus host disease and rheumatoid arthritis. Injection of MSC can be a potential therapy for juvenile idiopathic arthritis patients refractory to conventional therapies.

## Objectives

We tested the mechanisms of MSC immune modulation with a specific focus on the suppression of synovial fluid derived immune cells.

## Methods

We setup an *in vitro *culture system to test the suppressive capacity of MSC on both peripheral blood cells (PBMC) and synovial fluid cells (SFMC). We tested T cell proliferation and cytokine production of healthy controls and JIA patients. Furthermore, we investigated the induction or suppression of regulatory T cells (Treg) and Th17 cells as these cells have an important role in JIA pathogenesis and tested the effect of inflammatory cytokines and monocytes on MSC suppression.

## Results

MSC dose dependently suppressed both PBMC and SFMC, but SF T cells were less receptive towards MSC suppression. Tnfa and ifng were suppressed in both PBMC and SFMC, but IL-17 was not affected. In PBMC, but not SFMC, Th17 cell numbers were reduced by MSC and Treg numbers increased. We found no clear role for monocytes or inflammatory cytokines tnfa and ifng in activating the immune suppressive activity of MSC as published before.

## Conclusion

We conclude that MSC can suppress T cells derived from JIA synovial fluid. However, this suppression is reduced as compared to T cells derived from peripheral blood.

## Disclosure of interest

None declared.

